# MeCP2 Deficiency Disrupts Kainate-Induced Presynaptic Plasticity in the Mossy Fiber Projections in the Hippocampus

**DOI:** 10.3389/fncel.2019.00286

**Published:** 2019-07-03

**Authors:** Maria Laura Bertoldi, Maria Ines Zalosnik, Maria Carolina Fabio, Susan Aja, German A. Roth, Gabriele V. Ronnett, Alicia L. Degano

**Affiliations:** ^1^Departamento de Química Biológica Ranwel Caputto, Facultad de Ciencias Químicas, Córdoba, Argentina; ^2^Centro de Investigaciones en Química Biológica de Córdoba (CIQUIBIC), CONICET, Universidad Nacional de Córdoba, Córdoba, Argentina; ^3^Instituto de Investigaciones Médicas Mercedes y Martin Ferreyra (INIMEC), CONICET, Córdoba, Argentina; ^4^Center for Metabolism and Obesity Research, Johns Hopkins Medicine, Baltimore, MD, United States; ^5^Department of Neuroscience, The Johns Hopkins University, School of Medicine, Baltimore, MD, United States

**Keywords:** MeCP2, presynaptic plasticity, neurogenesis, activity-dependent gene expression, autism

## Abstract

Methyl cytosine binding protein 2 (MeCP2) is a structural chromosomal protein involved in the regulation of gene expression. Mutations in the gene encoding MeCP2 result in Rett Syndrome (RTT), a pervasive neurodevelopmental disorder. RTT is one of few autism spectrum disorders whose cause was identified as a single gene mutation. Remarkably, abnormal levels of MeCP2 have been associated to other neurodevelopmental disorders, as well as neuropsychiatric disorders. Therefore, many studies have been oriented to investigate the role of MeCP2 in the nervous system. In the present work, we explore cellular and molecular mechanisms affecting synaptic plasticity events *in vivo* in the hippocampus of MeCP2 mutant mice. While most studies addressed postsynaptic defects in the absence of MeCP2, we took advantage of an *in vivo* activity-paradigm (seizures), two models of MeCP2 deficiency, and neurobiological assays to reveal novel defects in presynaptic structural plasticity in the hippocampus in RTT rodent models. These approaches allowed us to determine that MeCP2 mutations alter presynaptic components, i.e., disrupts the plastic response of mossy fibers to synaptic activity and results in reduced axonal growth which is correlated with imbalanced trophic and guidance support, associated with aberrant expression of brain-derived neurotrophic factor and semaphorin 3F. Our results also revealed that adult-born granule cells recapitulate maturational defects that have been only shown at early postnatal ages. As these cells do not mature timely, they may not integrate properly into the adult hippocampal circuitry. Finally, we performed a hippocampal-dependent test that revealed defective spatial memory in these mice. Altogether, our studies establish a model that allows us to evaluate the effect of the manipulation of specific pathways involved in axonal guidance, synaptogenesis, or maturation in specific circuits and correlate it with changes in behavior. Understanding the mechanisms underlying the neuronal compromise caused by mutations in MeCP2 could provide information on the pathogenic mechanism of autistic spectrum disorders and improve our understanding of brain development and molecular basis of behavior.

## Introduction

Rett syndrome (OMIM #312750) is one of the few ASDs of monogenic origin that results in mental retardation, motor dysfunction, seizures, and features of autism. In 1999, the main cause of RTT was shown to be mutations in the MeCP2 (Amir et al., 1999). This protein is a member of the family of methyl-CpG binding proteins that bind to gene promoters and regulate their expression (Nan et al., 1997). Alterations in this protein have been found in patients with learning disorders and associated neuropathologies, suggesting that this protein plays an important role in the development and maintenance of neuronal circuits. Understanding the pathological mechanisms that lead to this syndrome would be of great importance for knowing the bases of these disorders and for promoting the development of therapies.

Numerous studies in animal models of RTT indicate that MeCP2 contributes to the formation and maintenance of neuronal connectivity; MeCP2 deficiency affects neuronal maturation (Matarazzo et al., 2004; Palmer et al., 2008), axonal and dendritic morphology (Belichenko et al., 2008; Chapleau et al., 2009), axonal guidance events (Degano et al., 2009), regulates synapse formation and function, as well as synaptic plasticity (Asaka et al., 2006). Although RTT is considered a neurodevelopmental disorder, more recent studies have revealed that MeCP2 is also critical for the maintenance of mature neural networks and global cerebral anatomy during stages of postnatal brain development and in the adult brain (Ballas et al., 2012).

Importantly, it has been demonstrated that *in vitro* MeCP2 regulates gene expression induced by neuronal activity; activity increases MeCP2 phosphorylation (Chen et al., 2001; Martinowich et al., 2003), which leads to dynamic interaction with specific co-repressors, for instance NCoR (Lyst et al., 2013), modulating the expression of target genes like BDNF. Although there is strong evidence that MeCP2 mediates activity-dependent responses *in vivo* (Zhou et al., 2006; Ebert et al., 2013), few studies have explored the consequences of MeCP2 mutations on synaptic plasticity, after *in vivo* synaptic stimulation.

Furthermore, the vast majority of the studies in the field have been focused on the consequences of MeCP2 mutation/deficiency on postsynaptic components of neuronal circuitries, i.e., dendritic morphology and arborizations, as well as density and morphology of dendritic spines (Chapleau and Pozzo-Miller, 2008). This was reasonable, as early evidence from post-mortem studies in RTT patients supported the hypothesis that this was a disorder of dendritic refinement (Chahrour and Zoghbi, 2007). Therefore, most efforts have addressed the role of MeCP2 on dendritic function and development, while the implication of MeCP2 on the development and function of presynaptic components (axonal projections), also essential for proper neural connectivity, remains relatively unexplored. To this end, using the olfactory system as a neurodevelopmental model, we previously identified a novel function for MeCP2 in axonal guidance processes during the establishment of neuronal circuits, including axonal trajectory, axon fasciculation, pruning, and axonal targeting (Degano et al., 2009). Moreover, defects in these processes were more evident when synaptic activity was stimulated (in this case, after odorant exposure) (Degano et al., 2014).

**FIGURE 1:**
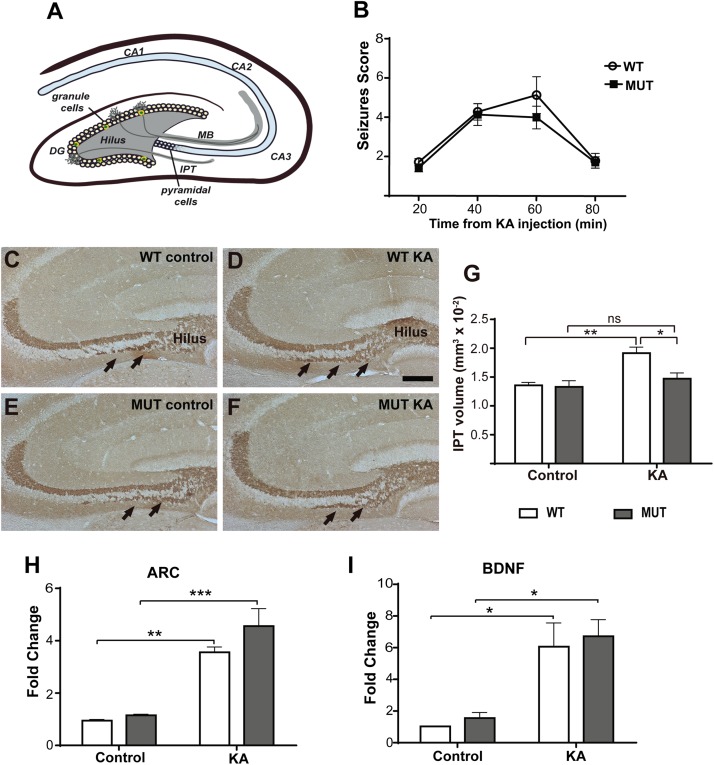
KA-induced seizures failed to increase IPT size in a mouse model of MeCP2-308 mutation. **(A)** Hippocampal MF-CA3 circuit. The MF consist of the axons of the granule cells of the DG that project through the main tract (MT) in the lucid stratum or through the IPT located mainly in the stratum oriens. **(B)** Seizures activity over time after KA administration in MeCP2-308 MUT model. No differences in the development or severity of seizures were observed between WT and MUT mice in response to KA (*n* = 5 mice per experimental group, two-way ANOVA *p* = 0.7023, followed by Tukey’s HSD test). **(C**–**F)** Representative images of IPT stained with anti-Synaptoporin in coronal hippocampal sections; IPT is indicated by black arrows. Two weeks after KA injection, WT mice **(D)** but not MUT mice **(F)** showed increased IPT size, in comparison with WT and MUT controls **(C,E)**. Scale bar: 200 μm. **(G)** Quantitative analysis of IPT volume in WT and MeCP2-308 MUT mice. KA-treated WT mice showed an increase in the IPT related to the controls; however, KA-treated MUT mice showed similar IPT size as MUT control. *N* = 4 mice per experimental group. **(H,I)** KA-induced gene expression: BDNF and Arc expression were determined by real-time RT-PCR from total hippocampus. Six hours after KA injection, a significant increase in the expression of the early activation genes Arc **(H)** and BDNF **(I)** was observed. Fold change was calculated in reference to the WT control group, which was normalized to 1. Results are represented as the ratio between the relative amount of the gene of interest and GAPDH. *N* = 4 mice per group. cDNA was prepared individually for each mouse and real-time RT-PCR reactions were run in duplicates for each mouse. Data analysis: two-way ANOVA followed by Tukey’s HSD test. ^*^p < 0.05; ^∗∗^*p* < 0.01; ^∗∗∗^*p* < 0.001.

The mouse hippocampus, a structure of great relevance in the pathophysiology of RTT (Chahrour and Zoghbi, 2007), displays two main events of presynaptic structural plasticity: adult neurogenesis at the DG, and dynamic changes in the size of the IPT, formed by granule cells MF (Figure 1A). Both forms of structural plasticity in the adult hippocampus correlate with improved performance in hippocampal-dependent learning tasks, and they are closely related to each other (Römer et al., 2011). It has been shown that exposure to an enriched environment or the induction of seizures activity favors adult neurogenesis in DG (Parent et al., 1997; Gray and Sundstrom, 1998; Lindvall et al., 2002), as well as the increase in the size of the IPT (Römer et al., 2011). Therefore, we proposed to use mouse models of RTT and a paradigm of seizures induction to investigate the cellular and molecular mechanisms involved in activity-dependent plasticity *in vivo* from the perspective of pre-synaptic components in the hippocampus from MeCP2-mutant mice.

In the present study, we report that inducing seizure activity in MeCP2-mutant animals reveals a novel role for MeCP2 in the structural plasticity of the MF-CA3 circuit. The lack of MeCP2 interferes with activity-dependent structural presynaptic plasticity in the hippocampus, affecting axonal growth/remodeling, the maturation of adult born neurons, as well as BDNF and Sema3F signaling. Likewise, we show that these plasticity defects correlate with a reduced performance in a spatial learning test (Barnes maze).

We propose that this is a model to study the causal relationships between structural alterations in the hippocampus and cognitive alterations present in mouse models of RTT. These studies may lay the foundations to establish a system that allows evaluating the effect of manipulating specific pathways involved in neural connectivity in a specific circuit and correlating it with changes in behavior. Moreover, these results may serve useful for approaching *in vivo*, in real-time studies for understanding the molecular basis of behavior in several neurodevelopmental or neurocognitive disorders.

## Materials and Methods

### Mice

We used two RTT mouse models for these studies. (1) MeCP2-308 model (MUT), these animals carry a premature stop codon at amino acid 308, generating a truncated MeCP2 protein that lacks the C-terminal region (B6.129S-Mecp2 <tm1hzo>/J, Stock 005439, The Jackson Labs) (Shahbazian et al., 2002) and (2) MeCP2-Bird model (KO): these mice carry a conditional deletion in exons 3 and 4 of the Mecp2 gene causing the absence of this protein, starting at embryonic stages (B6.129P2(C)-Mecp2 <tm1.1Bird>/J, Stock 003890, The Jackson Labs) (Guy et al., 2001). Both colonies were maintained in a C57BL/6J background. All the experiments were performed using only hemizygous Mecp2 males (Mecp2 MUT or Mecp2 KO) and their corresponding WT male littermates as control. Animal procedures were fully reviewed and approved by the local animal committee (School of Chemistry, National University of Córdoba, Protocol 2018-832), which follows guidelines from the National Institute of Health.

### *In vivo* Activity Paradigm

Male WT, KO, and MUT mice were genotyped following protocols provided by The Jackson Labs. Each mouse was administered with a single intraperitoneal (i.p.) injection of kainic acid (KA, 20 mg/kg, Sigma). As a control, the same volume of sterile PBS was injected (McLeod et al., 2013). Considering there are no reports of KA injection in the MUT and in order to provide similar seizure stimulus in WT and MUT mice, we performed several pilot experiments before selecting an adequate age for these experiments. At 6 weeks, the MUT mice were found to be resistant to KA, no convulsions were observed, nor an increase in the expression of activity-induced genes in hippocampus (i.e., BDNF or Arc measured by real-time PCR, data not shown). Conversely, 9-weeks-old MUT males showed similar kinetics and seizure activity as their respective WT littermates (Figure 1B). Therefore, since we observed differential sensitivity to KA at different ages in the MeCP2-308 model respect to WT littermates, we decided to use 9-weeks-old MUT males in our experiments for the purpose of using KA exposure as an *in vivo* stimulation paradigm. In the case of the KO, we used 6-weeks-old males, since the response to KA was already reported in the literature (McLeod et al., 2013). It is important to mention that the survival of the two mouse models is very different; male MUT mice live about a year (Shahbazian et al., 2002) while male KO mice survive about 10–12 weeks (Guy et al., 2001).

After KA injection, seizure response was recorded, which became evident after 20 min of injection. From the moment of KA injection, the animals were individually housed in separate boxes and monitored for several hours. The severity of the seizure response was graded according to the following scale: 0, similar to control; 1, increased respiratory rate; 2, in a state of “freezing” and with erratic contractions of the body; 3, straight and stiff tail with or without shaking; 4, the front legs begin to tremble; 5, straight and stiff tail along with tremor of front legs (once); 6, continuously shows straight and stiff tail along with front leg tremor (more than twice); 7, complete clonic tonic seizures, jumps; 8, death [adapted from McLeod et al. (2013)].

### Immunohistochemistry

Whole brains were harvested from WT and Mecp2 MUT or KO mice after cardiac perfusion with ice-cold PBS and 4% paraformaldehyde (PFA; Sigma), embedded with Cryoplast (Biopack, Argentina) and serial coronal cryo-sections (20 μm) were obtained. For each brain, 12 numbered slides were used and each one carried six serial sections of the hippocampus. Thus, a fragment of the dorsal region of the hippocampus extending 240 μm along the rostro-caudal axis was represented on each slide (Römer et al., 2011). For BrdU labeling and IPT staining, we used a peroxidase method (ABC System, Vectastain, Vector Laboratories) with biotinylated anti-mouse and anti-rabbit antibodies (1:200; Vector Labs). Fluorescence IHC was performed according to our established protocols (Degano et al., 2009). Primary antibodies include synaptoporin (Synpr; 1:1000; Synaptic Systems), βIII tubulin (TUJ1; Covance; 1:1000), bromodeoxyuridine (BrdU; 1:1000; Roche), GFAP (1:250; Sigma), NeuN (1:1000, LSBio), doublecortin (DCX; 1:200; Santa Cruz), and phospho-TrkB receptor (pTrKB; 1:250; Millipore). Secondary antibodies include biotin donkey anti-rabbit or mouse (1:200, Vector Labs). Cy3 donkey anti-rabbit IgG (1:500), Alexa 545 anti-goat (1:1000), Alexa 488 donkey anti-rabbit, and mouse IgG or chicken (1:1000) were used to facilitate double or triple labeling (Jackson ImmunoResearch). Data were obtained from at least four animals from 3 L per genotype per time point. Images were collected using either a Zeiss Axioskop with a digital camera (Axiocam; Zeiss) or an Olympus FV1000 scanning confocal microscope equipped with a Kr/Ar laser.

### Measurement of IPT Volume

Serial coronal sections were stained by IHC, using an antibody against the presynaptic vesicular protein: synaptoporin, enriched in MF (Singec et al., 2002). Images from hippocampus were obtained with an Axioplan microscope (Carl Zeiss) equipped with an Olympus XM10 camera. According to Römer et al. (2011), the IPT area was measured in each section, using the area measurement tool of the FIJI/Image J program (NIH). Then, the IPT volume was obtained for each slide, by multiplying the sum of the areas measured by the inverse of the sampling fraction (12) and by 20 (the thickness of the section in micrometers).

### Cell Counts

In order to label proliferating cells, WT and MUT mice were i.p. injected with BrdU (50 mg/kg, Sigma) in 0.01 M PBS. For assessing KA-induced neurogenesis, different cohorts of dividing cells were labeled in a single day. Thus, three doses of BrdU (6 h apart) were injected on the 6th day after KA induction, since it was reported that the highest proliferative activity in DG is detected at 1 week from seizure induction (Burns and Kuan, 2005; Römer et al., 2011). The animals were perfused 2 days after BrdU administration; coronal cryosections were prepared and processed for IHC using anti-BrdU antibodies. Sections were analyzed with an Axioplan microscope (Carl Zeiss) equipped with an Olympus XM10 camera. BrdU-positive cells were analyzed and counted along the rostral–caudal extension of the granule cell layer of DG. The resulting numbers were then multiplied by 12 to obtain the estimated number of total cells per granule cell layer of DG (Römer et al., 2011).

In order to study the maturation of adult-born granule cells of DG, different cohorts of progenitor cells were labeled during 3 consecutive days: 5th, 6th, and 7th days after KA administration (temporal window of high proliferative activity), injecting two doses per day, separated by 7 h. The animals were then perfused 4 weeks after the last BrdU dose, enough time for the new neurons to reach the characteristic morphology of mature granule cells (Burns and Kuan, 2005).

Coronal sections were subjected to triple labeling IHC using: anti-BrdU, to visualize new DG cells and both anti-DCX and anti-neuronal nuclear protein (NeuN) that allows identifying immature and mature neurons, respectively. Triple labeling images (BrdU, DCX, and NeuN) were acquired using Olympus Fluoview 1000 and 1200 confocal microscopes, using a 60× objective. Only BrdU-positive cells located in the granule cell layer of DG were included in the analysis. The markers colocalization was analyzed through the entire *Z*-axis of each BrdU-positive cell (covering the whole cell soma) in single optical planes of 0.7 μm thickness. Total BrdU-positive cells were counted and their colocalization was analyzed with the neuronal markers in order to calculate the percentage of new mature and immature neurons for each animal.

### Real-Time RT-PCR

Total RNA samples were prepared from five to six individual male mice per genotype per condition. Tissues were immediately frozen in liquid nitrogen and homogenized on dry ice. Total RNA was extracted with TRIzol reagent (Invitrogen) according to the manufacturer’s protocol. Genomic DNA was digested with 1 unit of DNase I (Invitrogen). cDNA was produced using the mMLV reverse transcriptase (Promega) following standard protocols (Degano et al., 2014). Real-time PCR was carried out on an iCycler (Bio-Rad) by using a reaction mixture with SYBR Green as the fluorescent dye (Applied Biosystems), a 1/10 vol of the cDNA preparation as template, and 250 nM of each primer (Degano et al., 2009, 2014). Primer sequences for BDNF exon IX was obtained from Li et al. (2012). The cycle used for PCR was as follows: 95°C for 180 s (1 time); 95°C for 30 s, 60°C for 30 s, and 72°C for 30 s (40 times); and 95°C for 60 s (1 time). Samples were subjected to a melting-curve analysis to confirm the amplification specificity. The change in fluorescence of SYBR Green dye was monitored in every cycle and the threshold cycle (CT) was calculated above the background for each reaction. For each cDNA sample, a ratio between the relative amounts of target gene and GAPDH was calculated to compensate for variations in quantity or quality of starting mRNA, as well as for differences in reverse transcriptase efficiency. The fold change in the target gene relative to the GAPDH endogenous control gene was determined by: fold change = 2^−Δ(ΔC_T_)^, where ΔC_T_ = C_T,target_ − C_T,GAPDH_ and Δ(ΔC_T_) = ΔC_T,KO_ − ΔC_T,WT_. RT-PCRs were run separately for each mouse in triplicate, and data were analyzed for statistical differences by two-way ANOVA using PRISM 6.0 software (GraphPad, San Diego, CA, United States).

### Barnes Maze

Barnes maze test was used to evaluate the presence of cognitive deficits in spatial learning and memory of MeCP2 MUT mice in comparison with WT littermates. For this purpose, a Barnes maze for mice was built following the specifications reported by other authors (Rosenfeld and Ferguson, 2014). It consisted of a 92-cm diameter circular platform constructed from a white PVC slab. Twenty holes of 5 cm diameter were made in the perimeter (separated from each other by 7.5 and 2 cm from the edge of the platform). A black exit box 20 cm long, 9 cm high, and 9 cm wide, with a ramp inside it (escape hole), was placed under one of the holes in the platform. The circular platform was located 1 m high from the ground and over it, 1 m away, was illuminated with two white light bulbs of 150 W each. Visual signs consisting of panels with simple colored shapes (squares, triangles, stars) were mounted as visual cues around the room where the test was performed. All of the sessions were recorded using a 12-megapixel camera placed above the platform.

The Barnes maze use is similar to the radial arm and Morris maze but without diet restrictions and with lower physical stress for mice. Inside the maze, mice are motivated to escape from the illuminated and open platform toward a dark, small, and recessed chamber (escape box). In this experiment, we worked with 12 weeks MUT and WT mice, using the protocol of O’Leary and Brown (2013) as reference. The test consists of four steps divided into 4 days:

^*^ Day 1. Habituation – It consisted in making the animal familiar with the environment, the platform, and the escape box. First, the animal was gently introduced into the escape box through the hole in the platform leading to it (“target” hole) and left there for 2 min. Then it was placed in the center of the platform for 30 s covered with a large transparent glass flask. After that period, the animal covered with the flask was guided to the target hole and the flask was located there for 3 min or until the animal enters the escape box. If after 3 min the animal did not enter the escape box, it was guided with the same flask to do so. Once inside the escape box, the animal was left there for 1 min and then it was returned to its usual cage.

^*^ Day 2. Training – Each animal was subjected to three trainings (4 min each), separated from each other by a half-hour break. Every training began with the lights off above the platform and placing the animal in the center of the platform covered by a non-transparent container. Then, the 4-min counting starts, the lights above the platform were turned on and the container that covers the animal removed, allowing it to freely travel the entire platform. When the animal entered the escape box (located in the same hole of the platform in all trainings), the lights were immediately turned off and the mouse was left there for 1 min. In the event that after 4 min the animal has not entered the box, it was guided to it and once inside the box, the lights were turned off again, and the mouse left there for 1 min. In each training we recorded: the percentage of time that the animal spent in each quadrant of the platform, the first quadrant to which the animal was directed when discovered, the number of total explorations of the holes of the platform (considering exploration when the animal pokes its head through one of the holes in the platform), the number of explorations in each quadrant, the number of correct explorations in the target hole, and the latency of entry to the escape box.

^*^ Day 3. Rest.

^*^ Day 4. Memory test – In this case each animal was subjected to a single exposure on the platform that lasted 2 min and in which the escape box was removed. Again, the test started with the lights off and placing the animal in the center of the platform covered by a dark container. When the 2-min count started, lights were turned on and the container covering the animal was removed, allowing it to freely travel the entire platform. Again, the same parameters were recorded here as in the trainings except for the latency of entry to the escape box.

### Statistical Analyses

All numerical analyses were performed using PRISM 6.0 software (GraphPad, San Diego, CA, United States). For all comparisons, two-way ANOVA was performed followed by Tukey’s *post hoc* test, when appropriate. Differences were considered significantly different at a *p* < 0.05.

## Results

### Kainic-Induced Seizures Failed to Increase the Size of the IPT in the Absence of MeCP2

To evaluate presynaptic structural plasticity in the absence of MeCP2 (i.e., adult neurogenesis and increase in the size of the IPT), we treated mice with KA to trigger seizures and robust neuronal activity, as shown by other authors (Ebert et al., 2013; Lyst et al., 2013). The animal models we use are from a C57BL6 background, which has a relatively high resistance to excitotoxic insults; therefore, they develop seizures in response to KA and neurogenesis is potentiated without leading to massive neuronal death (Schauwecker and Steward, 1997; Römer et al., 2011).

We generally used the MeCP2-308 mouse model (MUT) for these studies. Some of the experiments were replicated using MeCP2 Bird mouse models. MUT mice and their respective WT littermates were i.p. injected with KA or with sterile PBS (control), so we established four experimental groups: WT control, WT + KA, MUT control, and MUT + KA. In order to provide similar seizure stimulus in WT and MUT mice, we performed several pilot experiments before selecting an adequate developmental stage for this experiments (see the section “Materials and Methods”). A single KA dose was injected in 9-week-old MUT/WT mice, and seizure activity was assessed. Figure 1 illustrates the average seizure activity at several time points after KA injection. MUT mice (Figure 1B) responded to KA with similar seizure activity as the WT, showing comparable kinetics and maximum average scores (MUT: 4 ± 1.29; WT: 5 ± 1.83; *p* = 0.702).

Two weeks after the KA injection, all animals were euthanized and perfused for morphometric analysis, according to Römer et al. (2011). These authors described that during this time window in C57BL/6 mice, the IPT reaches the greatest increase in volume in response to KA-induced seizures. Serial coronal cryosections of the dorsal hippocampus were subjected to IHC for MF labeling and the volume of the IPT was calculated (Römer et al., 2011). As expected, an increase in the IPT size was observed in the WT + KA group comparing with WT controls (Figures 1C,D,G). However, no change in the IPT size was observed between MUT control and MUT + KA mice (Figures 1E–G). Thus, after KA injection, only WT mice showed a significant increase in the IPT size [*F*(1,17) = 4,858; *p* = 0.0416].

To determine whether the lack of IPT growth in response to seizure activity in MeCP2 MUT mice was caused by defective neural activation in response to KA, the expression of activity-induced neuronal genes (Arc and BDNF) was evaluated. For this, new groups of mice from both RTT models were injected with KA and 6 h later, whole hippocampus were dissected from control and KA-injected MUT mice and WT littermates. The expression levels of Arc and BDNF were quantified by real-time RT-PCR. As shown in Figures 1H,I, MUT mice showed a significant increase in the expression of Arc and BDNF mRNA after 6 h of KA treatment in comparison with PBS-injected controls. Likewise, WT littermates showed an increase in Arc and BDNF mRNA levels in response to KA. Therefore, no significant differences were recorded between MUT mice and the WTs in terms of expression levels of Arc [*F*(1.12) = 1.228; *p* = 0.2895] and BDNF [*F*(1,10) = 0.0034; *p* = 0.9521] in response to KA stimulation. These results demonstrate that in MUT MeCP2 animals an increase in neuronal activity was induced in response to KA administration, similar in magnitude to that induced in WT animals.

**FIGURE 2:**
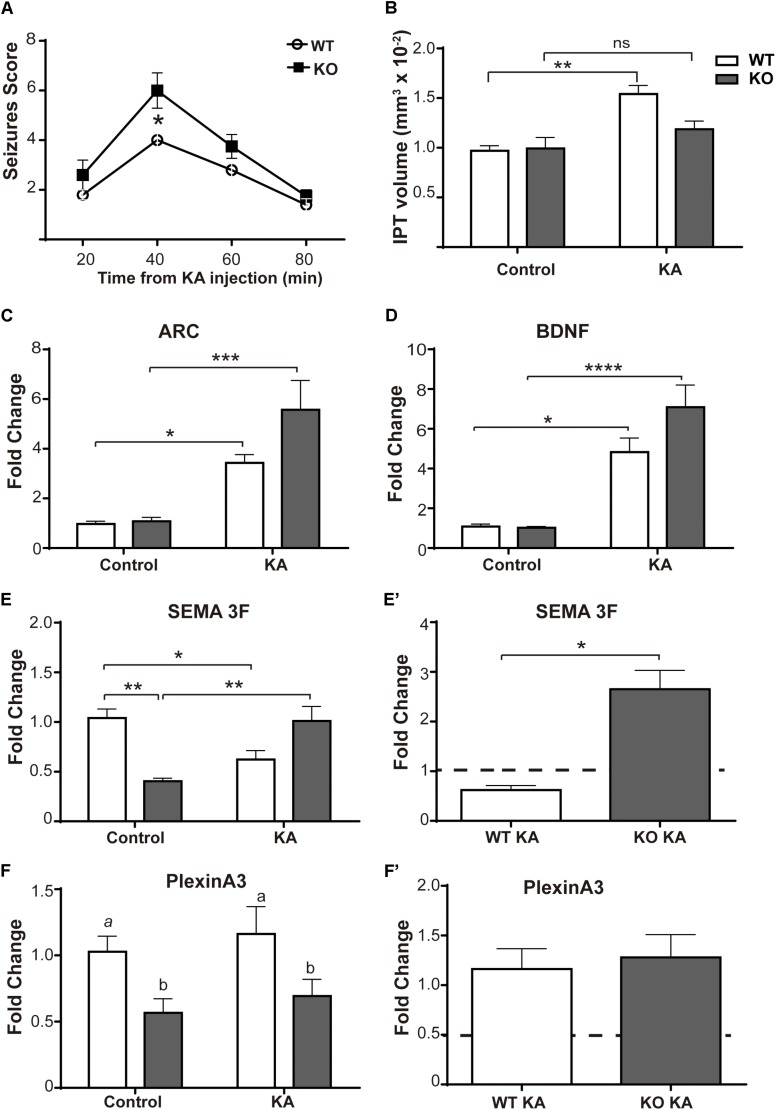
KA-induced seizures failed to increase IPT size in a mouse model of MeCP2 deficiency. **(A)** Seizures activity over time after KA administration. Six-week-old MeCP2-Bird KO mice elicit significantly higher seizures scores than WT littermates, indicative of greater sensitivity to KA. *N* = 4–5 mice per experimental group, two-way ANOVA, followed by Tukey’s HSD test, ^*^p < 0.05. **(B)** Quantitative analysis of IPT volume in WT and MeCP2-Bird KO mice. KA-treated WT mice showed an increase in the IPT volume with respect to controls; however, KA-treated KO mice showed similar IPT size as KO control. *N* = 4 mice per experimental group. **(C,D)** KA-induced neuronal gene expression: BDNF and Arc expression were quantified by real-time RT-PCR from total hippocampus. Six hours after KA injection, a significant increase in the expression of the early activation genes Arc **(C)** and BDNF **(D)** was observed. **(E,F)** Sema3F and Plexin A3 expression was assessed by real-time RT-PCR from total hippocampus. Two weeks after KA-seizures induction, a significant decrease in Sema3F was observed in KA-treated WT mice related to WT controls, whereas an opposite response was registered between KA-injected KO mice and KO controls. Basal Sema3F levels in KO mice were significantly lower than in the WT littermates **(E).** Regarding PlxnA3, no changes of expression were observed after KA treatment in WT or KO mice; however, PlxnA3 expression was significantly decreased in KO mice **(F)**. **(C–F)** Fold change was calculated in reference to the WT control group, which was normalized to 1. Results are represented as the ratio between the relative amount of the gene of interest and GAPDH. *N* = 5 mice per experimental group. cDNA was prepared individually for each mouse and real-time PCR reactions were run in duplicates for each mouse. Data analysis: two-way ANOVA followed by Tukey’s HSD test, ^*^p < 0.05; ^∗∗^*p* < 0.01; ^∗∗∗^*p* < 0.001. **(E′,F′)** In order to better evidence KA effect on each genotype, the expression levels generated by KA were re-calculated in relation to their respective control groups, WT or MUT. The dotted line on the *Y*-axis (*Y* = 1) represents the control values for each genotype. Mann–Whitney *t-*test with 95% confidence intervals, ^*^p < 0.05.

It is important to report that the results shown so far were reproduced using a mouse model of MeCP2 deficiency (MeCP2 Bird model, KO) (Figure 2). KO mice responded to KA more severely than WT mice, showing higher seizure scores (KO: 6 ± 0.707; WT: 4 ± 0.316; *p* < 0.05); and the levels of KA-induced Arc and BDNF expression in these animals were similar to the WTs (Figures 2A,C,D). Nevertheless, KO mice also failed to show an increase of the IPT after seizure activity (Figure 2B).

### Kainate-Induced Adult Neurogenesis and Survival of Adult-Born Neurons Are Not Affected in DG of MeCP2 Mutant Mice

**FIGURE 3:**
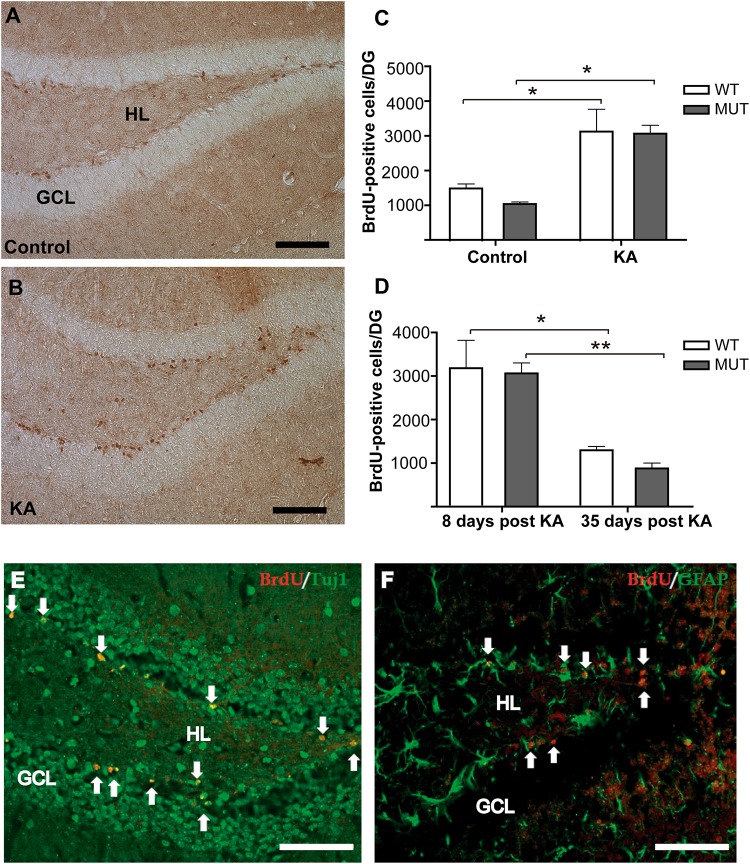
Seizures induce similar rate of adult neurogenesis in WT and MeCP2-308 MUT mice. **(A,B)** IHC for BrDU labeling. Eight days after KA injection, a strong increase in the number of BrDU-positive cells was observed in DG from KA-treated mice **(B)** compared with control littermates **(A)**. Scale bar: 50 μm. GLC, granule cell layer; HL, hilus. **(C)** BrDU-positive cells per DG in KA-treated and controls WT and MUT mice. A single seizure episode induces an increase in the number of BrDU-positive cells that was detected in both WT and MUT mice, respective to their controls. **(D)** Quantification of BrdU-positive cells by DG in WT and MUT mice injected with KA. A similar decrease in the number of BrdU-positive cells was observed in the WT and MUT mice between days 8 and 35 post KA-induced activity. **(E,F)** Double labeling IHC for BrdU and neuronal or astroglial markers in hippocampus 8 days after KA administration. **(E)** Double labeling of newborn DG cells (BrdU in red, white arrows) and Tuj1 (green) a neuronal marker. **(F)** Double labeling of newborn DG cells (BrdU in red, white arrows) and GFAP (in green) labeling astroglial cells in hippocampus. Scale bar: 50 μm. *n* = 3 mice per each experimental group. Data analysis: two-way ANOVA followed by Tukey’s HSD test, ^*^p < 0.05; ^∗∗^*p* < 0.01.

It has been shown that the axons of adult-born neurons in DG contribute mainly to the plasticity of the IPT tract (Römer et al., 2011). Likewise, it has been reported that there is a significant rise in the number of new granule cells in the DG after seizure activity (Parent et al., 1997; Gray and Sundstrom, 1998; Scott et al., 1998; Parent and Lowenstein, 2002; Römer et al., 2011), supporting the idea that adult neurogenesis could be related to the increase of the IPT size. Therefore, we quantified the rate of adult neurogenesis in DG after KA administration. Nine-week-old MUT mice and WT littermates received a single i.p. injection of KA or PBS; 6 days later we labeled several cohorts of cell progenitors by i.p. BrdU injections. Animals were sacrificed 2 days later (8 days after KA injection), the dividing cells were labeled by IHC, and BrdU-positive cells were counted along the rostral–caudal extension of the granule cell layer of DG. Eight days after KA injection, a strong increase in the number of BrdU-positive cells was observed in the DG of KA-treated mice compared to the controls (Figures 3A,B). However, there were no significant differences in the neurogenic response to KA between the two genotypes [*F*(1,8) = 0.2665; *p* = 0.6197]. A significant increase in the number of dividing cells was observed in both WT and MUT KA-treated mice in comparison with their respective controls (Figure 3C). In addition, no significant differences were found between both genotypes in the basal numbers of BrdU-positive cells per DG (WT control: 1464 ± 103, MUT control: 1034 ± 61) (*p* = 0.8169). To confirm that DG dividing cells were neurons, we performed double labeling IHC for BrdU combined with the neuronal marker tubulin β-III (Tuj1) or with an astroglial cell marker (GFAP). Most BrdU^+^ cells co-localized with the neuronal marker Tuj1, but not with GFAP. Figures 3E,F illustrate an example of this control, which was repeated for the samples analyzed. The data suggest that adult neurogenesis in DG is not affected by mutations in MeCP2, since a similar proliferative response was found in MUT and WT mice. However, it is possible that the survival of these new neurons is affected in the absence of MeCP2. For this purpose, we evaluated the proportion of KA-induced newborn DG cells (BrdU^+^) that remained 5 weeks after the induction of seizure activity. Figure 3D illustrates the number of BrdU^+^ cells per DG 8 days and 35 days after KA-induced seizures, when the new DG cells become mature neurons. At 35 days, BrdU^+^ cells from WT mice decrease about 60% and in MUT 71% respect to day 8; therefore, no significant differences were detected between the genotypes in terms of BrdU^+^ cell loss [*F*(1,9) = 0.2091; *p* = 0.5883]. Also, no differences were detected in the absolute number of BrdU^+^/DG cells between both genotypes at either 8 (*p* = 0.9987) or 35 (*p* = 0.8383) days after KA.

These observations suggest that the survival of the new DG neurons is not affected by MeCP2 mutation; therefore, we conclude that the lack of increase in the size of the IPT, registered 2 weeks after KA injection, was not due to a loss of new DG cells in MUT mice.

### Defective Maturation of Adult-Born Granule Neurons Is Recapitulated in Kainate-Treated MeCP2-Mutant Mice

**FIGURE 4:**
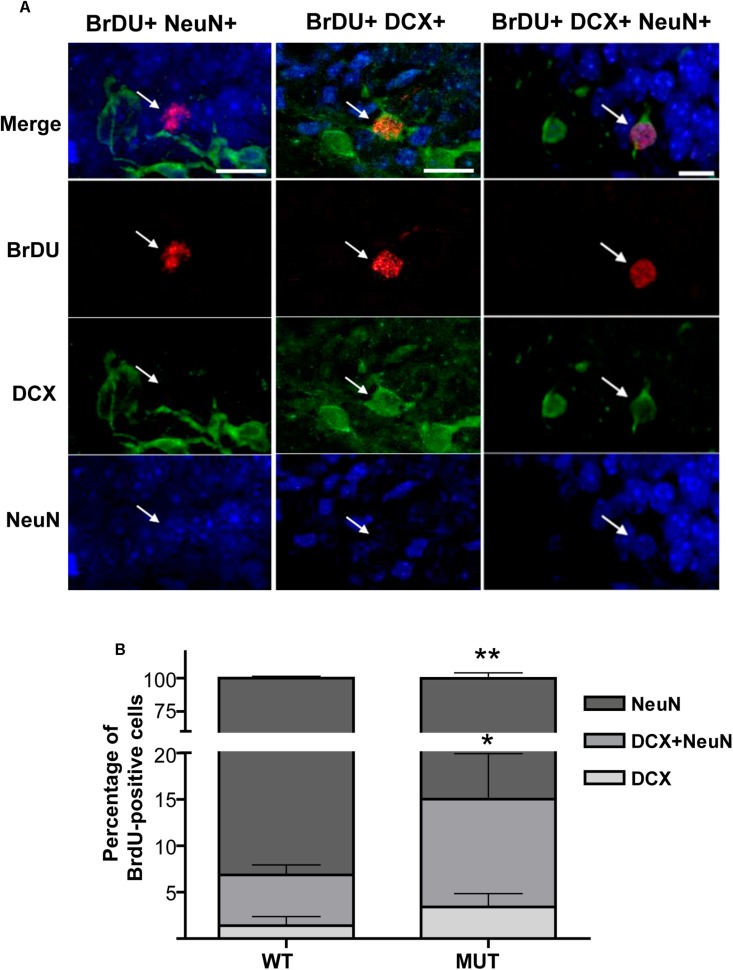
Defective maturation is recapitulated in MeCP2 mutant adult-born granule neurons. **(A)** Triple IHC labeling of BrdU-positive cells and neuronal markers NeuN and DCX. Examples of BrDU-positive neurons in intermediate state of maturation that co-express both NeuN and DCX (right panel). Example of a mature neuron that only expresses NeuN (left panel) and an immature neuron that only expresses DCX (middle panel). The vertical panels are unique optical planes showing the three fluorescence channels separately and their overlapping in the upper panel. Scale bar: 15 μm. **(B)** Percentages of BrdU-positive cells in different stages of maturation in WT and MUT mice exposed to KA. 5 weeks after seizures, WT mice showed a high percentage of mature new granule cells (90%), while MUT mice presented significantly less mature cells and more immature cells. Each bar represents the mean ± standard error (SEM) of BrdU-positive cells co-localizing with the corresponding markers, normalized to the total number of BrdU-positive cells in each mouse. Each bar is subdivided to indicate the mean percentage of BrdU-positive cells at different states of maturation. *n* = 4–5 mice per experimental group. Data analysis: two-way ANOVA followed by Sidak’s test, ^*^p < 0.05; ^∗∗^*p* < 0.01.

Previous work in the olfactory system and in the hippocampus from MeCP2-KO mice has reported a delay in neuronal maturation during early postnatal development, which is apparently compensated later on (Smrt et al., 2007; Palmer et al., 2008). Given that DG cells undergo adult neurogenesis, we wondered whether the new neurons of the adult organism recapitulate the defects shown in early postnatal development in a model of MeCP2 mutation. Therefore, we evaluated the percentage of maturation of adult born granule cells, taking advantage of the increase in adult neurogenesis induced by seizure activity. Because the numbers of BrdU-positive cells were extremely low in control individuals (injected with PBS), we report only the KA-treated groups. For this study, different cohorts of cell progenitors were marked by BrdU injections when the neurogenesis reached maximum levels (5th, 6th, and 7th day after KA injection), and the animals were perfused 4 weeks from the last BrdU dose. According to the literature, at that time adult-born DG neurons reach the characteristics of mature granule cells (Esposito, 2005). We performed triple immunofluorescence using three markers: anti-BrdU (dividing cells), anti-DCX as a marker of immature neurons, and the nuclear transcription factor NeuN, as a marker for mature granule cells (Esposito, 2005). All the BrdU-positive cells were counted and analyzed per animal sample, and we calculated the percentage of the adult-born neurons that reached maturity (BrdU^+^ NeuN^+^), the percentage of immature cells (BrdU^+^ DCX^+^) and at intermediate stage of maturation (BrdU^+^ DCX^+^ NeuN^+^). Figure 4A illustrates examples of double and triple labeling for BrdU-positive cells and the neuronal markers NeuN and DCX. Figure 4B shows significant differences between WT and MeCP2 MUT mice [*F*(2,21) = 13.73; *p* < 0.001]; the percentage of new DG cells that reached maturity was significantly lower in MUT mice (MUT_NeuN_: 86.39 ± 1.494%; WT_NeuN_: 93.13 ± 0.762%). In addition, MUT mice showed a higher proportion of both immature cells (MUT_DCX_: 3.85 ± 0.617%; WT_DCX_: 1.41 ± 0.481%) and cells in transit to maturation (MUT_DCX__+__NeuN__+_: 9.76 ± 1.521%; WT_DCX__+__NeuN__+_: 5.46 ± 0.537%). Thus, MUT adult-born granule cells show abnormal maturation; immature cells accumulate at expense of lower numbers of mature cells.

These results indicate that mice carrying a mutated MeCP2 protein (MUT) show defective neuronal maturation in the adult hippocampus, similar to what was observed during early development in a different mouse model (Smrt et al., 2007).

### Altered Expression of Activity-Induced Axon Guidance Molecules in the Context of MeCP2 Mutations

Using the olfactory system and MeCP2-deficient mice, we have demonstrated that axonal guidance defects were accompanied by alterations in the expression of Sema 3F and its receptor complex (PlexinA3 and Neuropilin-2). It has been demonstrated that this axonal guidance molecule regulates axonal growth, guidance, and targeting of hippocampal MF, acting as an inhibitor of axonal growth when combined with its receptor complex present in axons and inducing growth cone collapse (Bagri et al., 2003; Sahay et al., 2003; Riccomagno et al., 2012). Considering this background and the failure of the plastic response of IPT to seizure activity in MeCP2 MUT mice, we explored the Sema 3F pathway in our experimental conditions.

**FIGURE 5:**
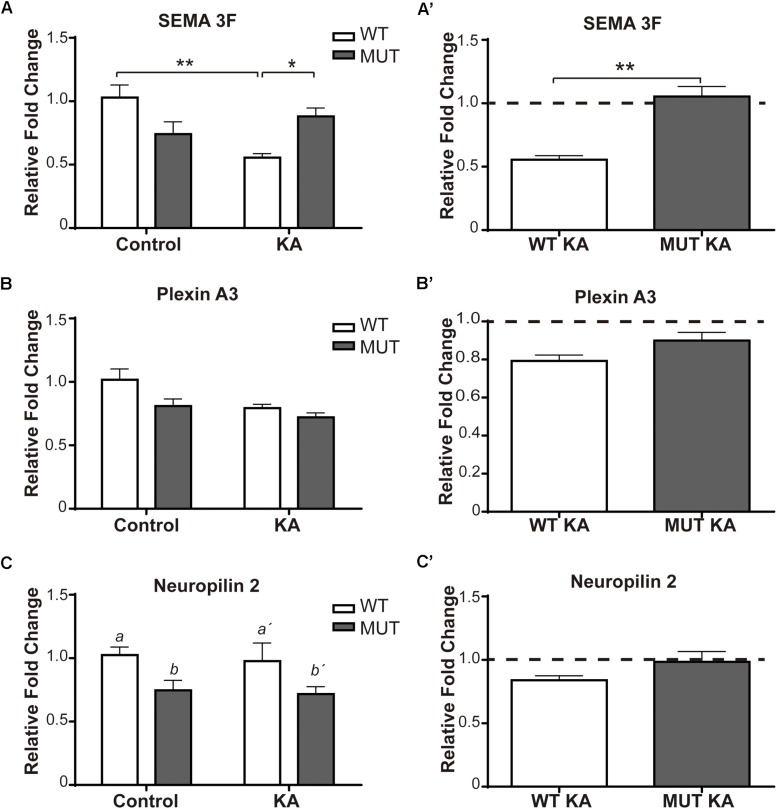
Kainic-induced Sema3F and receptors levels in WT and MeCP2-308 MUT mice. **(A–C)** Sema3F, Plexin A3, and Npn-2 expression were assessed by real-time RT-PCR from total hippocampus. Two weeks after KA-seizures induction, a significant decrease in Sema3F was observed in KA-treated WT mice related to WT controls, whereas no differences were registered between KA-injected MUT and MUT control mice **(A,A′)**. Regarding PlxnA3, no changes of expression were observed after KA treatment in WT or MUT mice **(B,B′)**; however, Npn-2 expression in MUT mice was significantly lower than WT mice, independently of treatment **(C,C′)**. **(A–C)** Fold change was calculated in reference to the WT control group, which was normalized to 1. Results are represented as the ratio between the relative amount of the gene of interest and GAPDH. *N* = 5 mice per experimental group. cDNA was prepared individually for each mouse and real-time PCR reactions were run in duplicates for each mouse. Data analysis: two-way ANOVA followed by Tukey’s HSD test, ^*^p < 0.05; ^∗∗^*p* < 0.01; ^∗∗∗^*p* < 0.001. **(A′**–**C′)** In order to better evidence KA effect on each genotype, the expression levels generated by KA were re-calculated in relation to their respective control groups, WT or MUT. The dotted line on the *Y*-axis (*Y* = 1) represents the control values for each genotype. **A′**–**C′:** Mann–Whitney *t*-test with 95% confidence intervals.

Methyl-CpG 2 binding protein 2 MUT mice and WT littermates were injected with KA or PBS and 2 weeks later the hippocampus of the different experimental groups were dissected. mRNA expression levels of Sema 3F gene and their two receptors (PlxnA3 and Npn-2) were quantified by real-time RT-PCR. Figure 5 shows the expression levels of the genes analyzed in MUT and WT animals.

Regarding Sema 3F (Figure 5A), considerable differences of expression were detected between WT and MUT mice [*F*(1,15) = 4.7; *p* < 0.05]. A significant decrease of Sema3F expression was observed in WT mice + KA compared to WT controls, while in MUT mice no change was registered between controls and KA-treated mice. Likewise, the WT + KA mice presented significantly lower levels of Sema 3F compared to the MUT KA. For PlxnA3 (Figure 5B), no significant differences were registered between both genotypes, neither between controls and KA-treated mice [*F*(1,14) = 1.327; *p* = 0.2687]. Although there were no changes in the expression of Npn-2 in response to KA treatment (Figure 5C), we found a significant lower expression in the basal levels of Npn-2 in MUT mice [*F*(1,14) = 8.072; *p* < 0.05].

Figures 2E,F show the expression of the Sema 3F and PlxnA3 in KO and WT animals of the MeCP2Bird model after KA-induced seizures. In this animal model, we observed robust differences in Sema 3F expression between WT and KO mice under basal conditions, and also after KA-induced seizures.

Summarizing these observations, the lack of increase in IPT size after KA treatment, in the context of MeCP2 mutations, was accompanied by a deregulation in the expression levels of Sema 3F which, in concert with the receptor complex, exerts an important chemo-repulsive effect on hippocampal axons (Riccomagno et al., 2012).

### BDNF Expression Induced by Neuronal Activity Is Deficient in Mecp2 Mutant Mice

**FIGURE 6:**
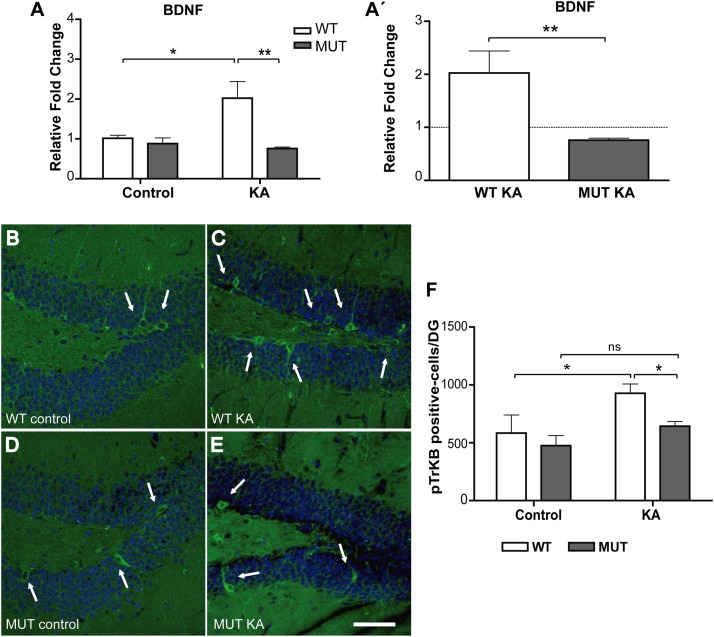
Kainic-induced BDNF expression is deficient in MeCP2-308 MUT mice. **(A,A′)** BDNF expression was assessed by real-time RT-PCR from total hippocampus. **(A)** Two weeks after KA-seizures induction, a significant increase in BDNF was observed in KA-treated WT mice related to WT controls, whereas no differences were registered between KA-injected MUT and MUT control mice. Fold change was calculated in reference to the WT control group, which was normalized to 1. Results are represented as the ratio between the relative amount of the gene of interest and GAPDH. *N* = 5 mice per experimental group. cDNA was prepared individually for each mouse and real-time PCR reactions were run in duplicates for each mouse. **(A′)** In order to better evidence KA effect on each genotype, the expression levels generated by KA were re-calculated in relation to their respective control groups, WT or MUT. The dotted line on the *Y*-axis (*Y* = 1) represents the control values for each genotype. Data analysis: **(A)** two-way ANOVA followed by Tukey’s HSD test, ^*^p < 0.05; ^∗∗^*p* < 0.01. **(A′)** Mann–Whitney *t*-test with 95% confidence intervals, ^∗∗^*p* < 0,01. **(B–E)** IHC- and pTrkB-positive cells quantification in DG. Two weeks after KA treatment, an increase in pTrkB-positive cells (white arrows) was observed in WT mice DG injected with KA **(C)** in comparison to KA-injected MUT mice **(E)** or control mice **(B,D)**. IHC labeling pTrkB (green) and Dapi (blue). Scale bar: 50 μm. **(F)** Quantification of pTrkB-positive cells per DG in WT and MUT mice injected with KA and control. A single seizure episode leads to an increase in the number of pTrkB-positive cells, detected 2 weeks after seizure activity only in WT mice. *n* = 3 mice per experimental group. Data analysis: two-way ANOVA followed by Tukey’s HSD test, ^*^p < 0.05.

In the hippocampus, neurotrophins levels are dynamically regulated by neuronal activity, and the modification of those levels can alter the growth and distribution of projections, and MF in particular. BDNF is normally expressed in most hippocampal neurons, including granule cells (Dieni et al., 2012; Isgor et al., 2015). This factor promotes the growth and orientation of granule cell axons *in vitro*, extends innervations of MF into CA3, and regulates its synaptic plasticity (Gómez-Palacio-Schjetnan and Escobar, 2008; Tamura et al., 2009; Römer et al., 2011; Schjetnan and Escobar, 2012). Also, it is now clear that MeCP2 is phosphorylated in response to synaptic activity, and that in this way, activity controls gene expression, i.e., BDNF production (Chen et al., 2001; Martinowich et al., 2003; Zhou et al., 2006). Although we have shown that BDNF levels increased similarly after 6 h of KA injection in both WT and MUT hippocampi (Figure 1I), we decided to evaluate BDNF expression after 2 weeks of treatment to determine whether BDNF expression correlates with the increase in IPT volume. As shown in Figure 6A, there was a significant difference between both genotypes in terms of BDNF expression [*F*(1,13) = 10.61; *p* < 0.01]. WT animals showed a moderate but significant increase in BDNF expression, 2 weeks after injection with KA. In contrast, in MUT mice BDNF expression remained unchanged between the control and the KA-treated group. Also, BDNF levels in MUT+KA animals were markedly lower than those of WT + KA mice (Figure 6A).

Considering that the effects of BDNF are largely mediated through its interaction with and the activation of the TrkB receptor, we proceeded to confirm our result by assessing the number of cells that express the phosphorylated TrkB receptor, as an indicator of BDNF action *in vivo* (Li et al., 2012; Helgager et al., 2014). For this, cryosections from the experimental groups were perfused after 2 weeks of KA treatment, when WT animals showed an increase in BDNF levels and in the size of the IPT in response to KA. Using IHC, we labeled pTrkB-positive cells and it became clear that the area with higher numbers of pTrkB-positive cells was the granule cell layer of the DG (Figures 6B–E). We found a significant difference between WT and MUT mice at 2 weeks after KA administration [*F*(1,8) = 11.58; *p* < 0.01]. While WT + KA showed a significant increase of pTrkB-positive cells in comparison with the controls, no significant differences were observed between treatments in MUT mice (Figure 6F). Again, the numbers of pTrkB-positive cells from the WT + KA were significantly higher than those of the MUT + KA.

Altogether, our results indicate that in WT animals, the growth of the IPT, detected at 2 weeks after KA seizure induction, is accompanied by an increase of BDNF expression. Conversely, no increase in the size of IPT is observed in MUT mice, which correlates with deficient BDNF expression in response to KA injection.

### Mecp2 Mutant Mice Present Defects in Spatial Memory Evaluated Through the Barnes Maze Test

Given the defects found in activity-dependent plasticity of MF (IPT) in the dorsal region of MeCP2 MUT hippocampus and considering that this circuitry is mainly involved in the processing of spatial memory (Crusio et al., 1987; Crusio and Schwegler, 2005), we decided to assess spatial learning and memory abilities in these mice through their performance in the Barnes maze. It is estimated that, through successive training sessions, the improvement in performance to locate a hidden box to escape from aversive stimuli allows assessing learning processes and spatial memory in rodents (Rosenfeld and Ferguson, 2014). Considered less stressful for mice than water maze (Morris), the Barnes maze consists of a relatively simple design of a circular platform with several holes equally spaced around the perimeter edge. While one hole leads to an escape cage, the rest are blind. Slightly aversive stimuli (i.e., bright lights) provide the motivation to locate the escape cage, which is also flanked by visual cues (O’Leary and Brown, 2013). Therefore, MUT mice and their WT littermates were evaluated for spatial learning and memory according to their performance in the Barnes maze test.

#### Learning

**FIGURE 7:**
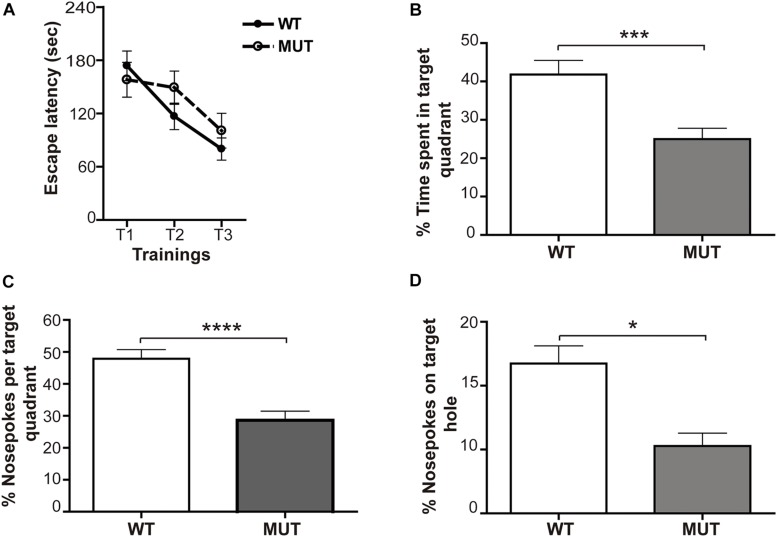
Mecp2-mutant mice show defective spatial memory evaluated through the Barnes maze. **(A)** Learning: the acquisition was reflected by lower escape latencies along the trainings. For both experimental groups, a similar decrease in the escape latency time (measured in seconds) throughout the successive trainings was observed. *n* = 16-19 mice per experimental group. Data analysis: two-way ANOVA followed by Tukey’s HSD test. **(B–D)** Memory: relative percentage of time spent and number of snouts made by the animals in the target quadrant and in the escape hole during the 2 min of the final memory test. **(B)** Percentage of time spent in target quadrant for WT and MUT mice. The analysis shows that WT mice spent significantly more time in the target quadrant than MUT mice. Percentage of snouts in the target quadrant holes **(C)** and in the escape hole **(D)** in relation to the total snouts performed in the holes of the platform in the 2 min of the final memory test. Both the percentage of snouts in the “target” quadrant **(C)** and in the escape hole **(D)** was significantly higher in WT mice when compared with the MUT. *n* = 16–19 mice per experimental group. Data analysis: Mann–Whitney *t*-test with 95% confidence interval. ^*^p < 0.05; ^∗∗∗^*p* < 0.001; ^****^p < 0.0001.

The acquisition or learning of the escape box location was assessed by determining the escape latency during three successive training sessions; thus, the acquisition was reflected by lower escape latencies along the trainings. Interestingly, the first thing we noticed was that the percentage of MUT animals that did not learn was almost 20% higher than the WT (WT = 9.52% mice did not learn the task; MUT = 27.3% did not learn the task); “non-learners” were those animals that did not enter the escape box in any of the three training sessions. However, when we evaluated the escape latency of the animals that did learn, no significant differences were found in the acquisition between the groups [*F*(6,117) = 0.744; *p* = 0.6150] (Figure 7A). We found that the average escape latency decreased similarly in both groups throughout the successive trainings. This observation suggests that, although a higher percentage of MUT mice failed to learn the task, in the groups that did learn, the learning process was similar to that of the WT littermates.

#### Memory

Two days after the acquisition phase, we performed a final test of retention or memory, consisting of a single 2-min exposure on the platform, after removal of the escape box. In this spatial preference test the animal that remembers the maze will spend more time in the “target” quadrant, i.e., the quadrant where the escape box was previously located (O’Leary and Brown, 2013). The data showed a significant difference between genotypes [*F*(1,36) = 14.81; *p* < 0.001] (Figure 7B). While WT mice spent about 40% of the time in the target quadrant, MUT mice only spent an average of 24% of the total time in that quadrant (Figure 7B). Since the average percentage of time that a normal mouse will spend randomly in each quadrant of the platform during the test is about 25%, we can conclude that the MUT mice showed a failure in retaining the task learned during the acquisition phase.

These results were complemented by quantifying the percentage of snouts made by the animals in the target quadrant and in the escape hole with respect to the total snouts in the holes of the platform during the 2 min of the final memory test. The statistics showed a marked difference between WT and MUT MeCP2 [*F*(1,38) = 22.7; *p* < 0.0001]. WT mice explored the target quadrant and the escape hole significantly more than MUT mice (Figures 7C,D). Altogether, these results indicate that MeCP2 MUT mice do not show defects in the spatial learning process measured with the Barnes maze; however, they exhibit a marked failure in the spatial memory evaluated by the same test.

## Discussion

In the present study, we report that inducing seizure activity in MeCP2 mutant animals reveals a novel role for MeCP2 in the structural plasticity of the MF-CA3 circuit. The lack of MeCP2 interferes with activity-dependent structural presynaptic plasticity in the hippocampus, affecting axonal growth/remodeling, the maturation of adult born neurons, as well as BDNF and Sema3F signaling. Likewise, we show that these plasticity defects correlate with a reduced performance in a spatial learning test (Barnes maze).

Two central events in presynaptic structural plasticity have been described in mice and rat hippocampus: adult neurogenesis and dynamic changes in the size of the IPT, formed by DG granule cell axons. Both the formation of new DG neurons and the anatomy of the IPT are dynamically regulated in response to neurogenic stimuli, such as exposure to an enriched environment or the induction of epileptogenic activity (Parent and Lowenstein, 1997; Gray and Sundstrom, 1998; Lindvall et al., 2002; Belichenko et al., 2008; Römer et al., 2011). Although environmental enrichment may be a better physiological stimulus, it is not possible to ensure that any given animal receive similar stimulation. In the present study, we decided to treat animals with KA in order to trigger seizures and generate robust neuronal activity *in vivo*, as a way to provide controlled stimulation conditions, leading to more homogeneous activity. We based our experimental design on several reports using KA injections in different Rett mouse models, considering that those studies have established most of the activity-dependent responses described in the field (Ebert, 2013).

Most of our studies were developed in the MeCP2-308 (MUT) model which better resembles the human pathology; these mice express a mutated protein, their clinical evolution is progressive, and they survive for over a year. This is important because it permits experiments at more advanced ages and also avoids the results may be masked by near-death physiological conditions, as occurs with most of the studies performed in MeCP2 KO models (survival: 8–11 weeks). The original characterization of this mouse model revealed that MeCP2 MUT animals develop spontaneous seizures from 5 months of age (Moretti et al., 2005). However, there were no reports regarding the induction of seizures activity using this model. Here, we performed KA injection experiments in WT and MUT mice of 6, 9, and 12 weeks of age. Interestingly, 6 weeks old-MUT mice injected with KA did not develop seizures nor showed an increase in the expression of BDNF and Arc, in contrast to their WT littermates (data not shown) and MeCP2-KO mice (Figure 2A). Conversely, 12-weeks-old MUT mice were acutely sensitive to the KA doses that were just effective in 12-weeks-old WT mice (data not shown). Lastly, 9-weeks-old WT and MUT mice injected with KA showed similar levels of seizures activity and increased expression of BDNF and Arc (Figures 1B,H,I). Thus, we found clear age-dependent differences in susceptibility to KA in MUT animals.

However, we observed no change in the IPT volume in 9-week-old MUT mice (Figure 1G), even though they showed increased neuronal activity in response to KA, similar in magnitude to that induced in WT animals (Figures 1B,H,I). These results suggest that the lack of IPT growth was not due to a defect in early neuronal activation in the hippocampus of MeCP2 MUT mice. We confirmed some of these results using another model MeCP2 Bird mice (KO). MeCP2 KO mice injected at 6 weeks of age showed greater sensitivity to KA than their WT counterparts (Figure 2A). This response is consistent with other author reports, using a different KO mouse model of MeCP2 (McLeod et al., 2013). When activity-induced gene expression was quantified after 6 h of KA, both WT and KO mice showed increased expression of BDNF and Arc (Figures 2C,D). However, only the WT mice of this model showed a significant increase in IPT size (Figure 2B), even though MeCP2 KO animals displayed higher seizures scores. Thus, using two different mouse models we show that even though similar seizure activity was recorded in every KA-injected animal and they show similar levels of hippocampal neural stimulation (measured as levels of activity-induced gene expression), no increase in the size of the IPT was detected in any of the models of MeCP2 deficiency (MUT or KO), indicating a deficit in the plastic IPT response to neuronal activity in the absence of normal MeCP2 function.

Since it has been demonstrated that the IPT size is dynamically influenced by an increase in neurogenesis (Römer et al., 2011), we evaluated adult neurogenesis in DG in response to KA. Our results showed that this process was not affected in MeCP2 MUT mice (Figures 3A–C), in agreement with previous work indicating that MeCP2 is not critical for neurogenesis (Smrt et al., 2007). The lack of IPT growth observed in MeCP2 MUT mice in response to KA would not be a consequence of a lower production of new granule cells in DG. However, it is possible that the survival of these new neurons is affected in the absence of MeCP2. In this sense, we observed that the proportion of new cells (BrdU^+^) that remain 5 weeks in the DG after the induction of seizures activity was similar in WT and MUT mice (Figure 3D); therefore, the survival of the new DG neurons seems not to be affected by MeCP2 mutation. These results are also in agreement with older studies that found no signs of neurodegeneration or neuronal death in these models of MeCP2 deficiency (Moretti et al., 2005, 2006).

Interestingly, although neurogenesis was not affected in the absence of MeCP2, the new DG neurons displayed deficits in their ability to transition to a mature state (Figure 4). Our results show that mice carrying a mutated MeCP2 protein (MUT) have defects in the maturation of new neurons in adults, in a manner similar to that observed during early development in other RTT models (Matarazzo et al., 2004; Smrt et al., 2007; Palmer et al., 2008). The increase in adult neurogenesis induced by neuronal activity allowed us to show that this defect is recapitulated in the adult animal. The delay in maturation of DG newborn granule cells in adult animals suggests that, although in response to synaptic activity a similar number of new neurons are generated in the absence of MeCP2, they would not normally integrate into the circuit. This finding could have important implications in hippocampal-dependent learning processes, such as spatial memory tests.

Regarding the structural plasticity of the IPT tract, it has been proposed that this is the result of the coordinated increase of trophic factors and the decrease of chemo-repellant molecules (Bermúdez-Rattoni and Rekart, 2007). The levels of neurotrophins in the hippocampus are regulated dynamically by neuronal activity and the modification of these levels can alter the growth and distribution of the MF. Numerous studies have implicated BDNF as a potent modulator in many aspects of neuronal development (Huang and Reichardt, 2001), as well as in synaptic transmission and plasticity (Poo, 2001; Cowansage et al., 2010; Lu, 2003). BDNF normally expressed in most hippocampal neurons promotes the growth and orientation of the axons of granule cells *in vitro* and regulates the synaptic plasticity of MF (Gómez-Palacio-Schjetnan and Escobar, 2008; Tamura et al., 2009; Schjetnan and Escobar, 2012). In addition, MeCP2 has been shown to be phosphorylated in response to synaptic activity and regulates gene expression, in particular the production of BDNF (Chen et al., 2003; Martinowich et al., 2003). Our results demonstrate that in both WT and MeCP2 MUT animals, BDNF levels increase similarly in response to KA, at 6 h post injection. However, when we determined BDNF expression and pTrKB (an *in situ* indicator of endogenous BDNF protein; Li et al., 2012) at 2 weeks post-KA, we found it remains high in WT animals, but not in the MUT mice (Figure 6F). Thus, the IPT growth detected in WT animals was accompanied by an increase in the expression of BDNF; while in MeCP2 MUT mice, the lack of growth correlated with a poor change in BDNF expression. These results indicate that although the signaling events involved in the early neuronal response to seizure activity seems unaffected in the absence of MeCP2 (Figure 1H), we do detect a deficit of long-term BDNF support required for driving the axonal growth of MF in response to synaptic activity.

Given the importance of this neurotrophin, and considering that MeCP2 binds to one of BDNF promoters and regulates its expression, many studies have proposed a critical role for BDNF in the pathogenesis of RTT. In this sense, a general reduction in BDNF mRNA levels has been described in MeCP2 KO animal models (complete lack of protein) (Chang et al., 2006; Wang et al., 2006; Kobayashi, 2009; Li and Pozzo-Miller, 2014). However, it is interesting to note that those changes were reported when MeCP2 KO mice were already fully symptomatic (and with a life expectancy of 1–3 more weeks). In this sense, we found no differences in hippocampal BDNF expression between WT and MeCP2 KO 6-weeks-old mice under either basal or KA-treated conditions (Figure 2D), suggesting that BDNF expression in the absence of MeCP2 may be either associated to age or secondary to symptoms; this issue has been already noticed in our previous work and also discussed by other authors (Degano et al., 2014; Li and Pozzo-Miller, 2014). In contrast, in the present work, we show that in the MeCP2-308 mouse model, MUT mice in basal conditions show no differences in mRNA levels for BDNF in the hippocampus at either 9 or 11 weeks of age (Figures 1I, 6A). This observation was also reported in older and symptomatic MeCP2-308 MUT animals (Moretti et al., 2006). In addition, we observed that both WT and MUT groups responded with a similar increase in BDNF expression at 6 h post-KA (Figure 1I), although this response was not maintained over time in the MUT animals (Figure 6A). These results suggest a more complex relationship, and further studies are needed to clarify the role of BDNF in the absence of MeCP2 and in RTT pathogenesis (Li and Pozzo-Miller, 2014). Paradigms of *in vivo* synaptic activation combined with circuit plasticity and molecular tools may help to elucidate these relationships.

On the other hand, a body of evidence suggests that semaphorins are critical determinants of axonal growth and targeting during the development of the nervous system (Pasterkamp, 2012). Mice deficient in Sema 3F, Npn-2, or in PlexinA3 showed robust hypertrophy of the IPT tract (Cheng et al., 2001; Bagri et al., 2003; Riccomagno et al., 2012). Therefore, considering our previous reports revealing axonal guidance and Sema 3F deficits in MeCP2 KO mice (Degano et al., 2009), we focused in evaluating the Sema 3F pathway in the context of MeCP2 mutation. This molecule is involved in the growth, guidance, and target of hippocampal MFs (Bagri et al., 2003; Sahay et al., 2003), acting as an inhibitor of axonal growth and inducing their collapse (Riccomagno et al., 2012). In this sense, the response of WT mice injected with KA (Figure 5A) was in line to previous reports, which showed that WT mice exposed to seizure activity (using either KA or pilocarpine) display a decrease in mRNA Sema 3F levels between the 1st and 2nd week after seizures in CA3, CA1, and DG (Barnes et al., 2003; Cai et al., 2016); this decrease overlapped with the formation of new synapses in the MF-CA3 circuitry (Okazaki et al., 1995; Barnes et al., 2003). Thus, the reduction of Sema 3F expression induced by KA in WT animals (low chemo-repulsion) would promote a permissive milieu for MF growth, contributing to the dynamic increase in the size of the IPT registered after KA-induced seizures (Römer et al., 2011). Since both MUT and KO MeCP2 mice failed to show a decrease of Sema3F levels in response to KA (Figures 2E,E′, 5A,A′), we suggest that in these animals a local inhibitory environment for axonal growth is operating, which could account for the lack of IPT volume increase in the absence of normal MeCP2 expression.

**FIGURE 8:**
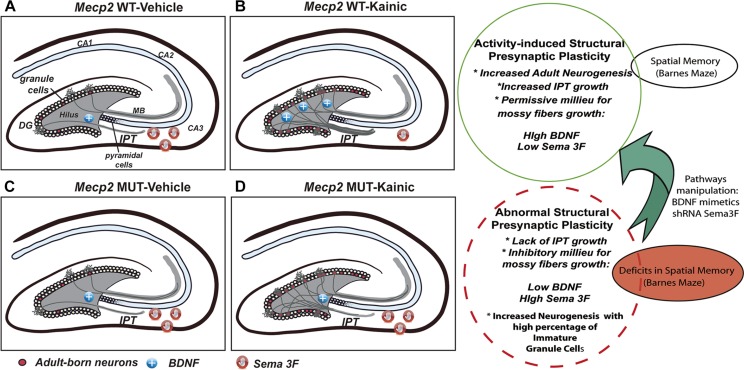
Schematic summary of the activity-induced presynaptic plasticity regulated by MeCP2. In WT mice, seizures activity increases the level of neurogenesis, accompanied with higher levels of BDNF and lower of Sema3F, allowing IPT to grow **(A,B)**. Conversely, MUT animals show abnormal activity-dependent presynaptic plasticity characterized by the lack of IPT growth which correlates with imbalanced trophic and guidance support (BDNF and Sema3F abnormal levels). Although activity do increases the neurogenesis in MUT mice, adult born granule cells show developmental restraining and recapitulate maturational defects **(C,D)**. These results are in line with deficits in spatial memory observed in MUT mice. This study set the basis to establish a model to evaluate the effect of manipulating specific pathways involved in axonal guidance, synaptogenesis, and neuronal maturation to correlate with changes in behavior.

It has been suggested that the process of structural plasticity shown by MF in response to synaptic activity involves a tightly regulated balance between growth factors and axonal guidance molecules (Bermúdez-Rattoni and Rekart, 2007). Here we report that an imbalanced expression of those crucial players might be responsible for the lack of MF growth in the absence of MeCP2 (Figure 8). Thus, after seizure activity, MeCP2 MUT and KO mice display an increase of the chemorepellant Sema 3F, which in concert with a deficient expression of the growth factor BDNF would generate an inhibitory environment for axonal growth.

In this study, we did not analyze the dendritic components of granule DG cells. However, in contrast to postmortem studies in RTT patients (Armstrong et al., 1995), the brains of symptomatic MeCP2-308 MUT mice did not show defects in dendritic arborizations in frontal cortex nor in the density of synapses or dendritic spines in CA1 area of hippocampus (Moretti et al., 2006). In addition, electrophysiological studies suggest that synaptic dysfunction precedes clinical symptoms; these were manifested as an increase in synaptic transmission and decreased LTD in the collateral synapses of Schaffer in this animal model (Moretti et al., 2006). Further studies using this animal model of MeCP2 mutation may define whether axonal components are the main substrate of these synaptic defects.

Presynaptic structural plasticity events have been correlated with improvements of performance in hippocampal-dependent learning tests (Römer et al., 2011). The considerable plasticity of the IPT has been observed early (Schwegler et al., 1990), and abundant evidence demonstrated the positive correlation between IPT size and animal performance in a great variety of behavioral tests of spatial memory (Lipp et al., 1988; Schwegler et al., 1990; Schöpke et al., 1991; Bernasconi-Guastalla et al., 1994; Laghmouch et al., 1997; Crusio and Schwegler, 2005; Delprato et al., 2015). Interestingly, deficits in radial maze performance were also associated with smaller IPT in a mouse model of Fragile-X Syndrome (Mineur et al., 2002). Therefore, we decided to evaluate spatial learning and memory of MeCP2 MUT mice through their ability to navigate the Barnes maze; it is estimated that the improvement in performance to locate an escape box to aversive stimuli, through successive training sessions, allows assessing learning processes and spatial memory in rodents. Previous studies showed that MeCP2-308 MUT mice (>20 weeks of age = fully symptomatic) show learning and memory deficits in several hippocampal-dependent behavior paradigms (Morris water maze, fear contextual conditioning test, and long-term social memory) (Moretti et al., 2005, 2006). Our results indicate that earlier in development (12-weeks-old), 27% of MeCP2-308 MUT mice failed to learn the location of the exit box, compared with 10% of WT littermates. Interestingly, the MUT animals that learned the task had similar performance as the WTs during the trainings. Conversely, these MUT animals displayed clear spatial memory deficits, as they showed a marked failure in the retention of the acquired information evaluated by the same test (Figures 7B–D). It is important to emphasize that in this work we also revealed that failure in granule cells maturation recapitulates in each round of neurogenesis in the adult hippocampus, adding to the long-term circuitry dysfunction (Figure 4). Although it has not been demonstrated that memory *per se* is altered in patients, cognitive abnormalities are present in individuals with MeCP2 mutations, supporting the concept that the results obtained with the MeCP2-308 animal model would represent a correlate of cognitive deficits in RTT (Moretti et al., 2006).

Alterations of synaptic and axonal refinement have been implicated in the etiology of neurological diseases (Lewis and Levitt, 2002; Johnston, 2004; Pardo and Eberhart, 2007). Likewise, hippocampal MF synapses are important for spatial memory formation and consolidation (Nicoll and Schmitz, 2005; Bischofberger et al., 2006). Spatial learning and environmental enrichment result in an increase in the number, size, and complexity of the MF (Ramírez-Amaya et al., 2001; Galimberti et al., 2006). In addition, the IPT presents a dramatic reorganization after induction of seizures (Cavazos et al., 1991). While the molecular pathways underlying the activity-dependent remodeling of MFs should still be determined, neurotrophins such as NGF and BDNF may play a role in the remodeling of MF in the normal brain, since their levels are increased with seizures activity (Zafra et al., 1990). Axonal guidance molecules including semaphorins, plexins, and neuropilins can also help to reconfigure MF after changes in activity levels (Holtmaat et al., 2003; Suto et al., 2010).

In light of the present studies, we propose that activity-dependent axonal defects and alterations in neurotrophins and guidance molecules signaling could contribute to the neurological defects present in models of RTT syndrome. Likewise, in this animal model we found a correlation between the structural plasticity of MF and the spatial memory response. This offers a powerful tool for studies on the consequences of MeCP2 mutation, since changes in a relatively simple neural circuit could be connected to changes in spatial memory defined from an animal behavioral test. Based on these studies, we establish a model that would allow to evaluate the effect of the manipulation of specific pathways involved in axonal guidance/synaptogenesis/neuronal maturation in a specific circuit in correlation with changes in spatial memory (Figure 8). This strategy may enable a better understanding of the factors that control synaptic activity-dependent remodeling in the normal brain and in disorders of connectivity, such as autism-related disorders.

## Data Availability

All datasets generated for this study are included in the manuscript and/or the supplementary files.

## Ethics Statement

Animal procedures were done in accordance with our Institutional Animal Care and Use Committee (IACUC from School of Chemistry, National University of Córdoba), which follows guidelines from the National Institute of Health.

## Author Contributions

MB, MZ, and MF contributed to the acquisition, analysis, or interpretation of data for the work. SA, GVR, and GAR contributed to key techniques and critically revised the work for important intellectual content. AD contributed to the conception and design of the study, and wrote the first draft of the manuscript. All authors contributed to the manuscript revision, read, and approved the submitted version.

## Conflict of Interest Statement

GVR is currently employed by company Janssen Research & Development, LLC. The remaining authors declare that the research was conducted in the absence of any commercial or financial relationships that could be construed as a potential conflict of interest.

## Abbreviations

ASDs: autism spectrum disorders
BDNF: brain-derived neurotrophic factor
DG: dentate gyrus
GFAP: glial fibrillary acidic protein
IPT: hippocampal infrapyramidal tract
MeCP2: methyl-CpG 2 binding protein 2
MF: mossy fibers
RTT: Rett syndrome
Sema3F: semaphorin 3F
TrkB: tropomyosin-related kinase B.
